# Design of a protein tag and fluorogenic probe with modular structure for live-cell imaging of intracellular proteins†
†Electronic supplementary information (ESI) available. See DOI: 10.1039/c5sc02351c


**DOI:** 10.1039/c5sc02351c

**Published:** 2015-09-30

**Authors:** Yuko Kamikawa, Yuichiro Hori, Kazuo Yamashita, Lin Jin, Shinya Hirayama, Daron M. Standley, Kazuya Kikuchi

**Affiliations:** a Graduate School of Engineering , Osaka University , Osaka 565-0871 , Japan . Email: kkikuchi@mls.eng.osaka-u.ac.jp ; Fax: +81-6879-7875; b IFReC , Osaka University , Osaka 565-0871 , Japan; c JST , PRESTO , Osaka 565-0871 , Japan

## Abstract

Conditional fluorescence imaging is a powerful technique for precise spatiotemporal analysis of proteins in live cells upon administration of a synthetic probe. To be applicable to various biological phenomena, probes must be membrane-permeable, have a high signal-to-noise ratio, and work quickly. To date, few probes meet all of these requirements. Here, we designed a fluorogenic probe (AcFCANB) that could label intracellular proteins fused to the photoactive yellow protein (PYP) tag in live cells within 30 min and used it to image heterochromatin protein 1 localization in nuclei. AcFCANB is based on a modular platform consisting of fluorophore, ligand and quencher. We accelerated the labeling reaction by strategic mutations of charged residues on the surface of PYP. A simple model based on molecular dynamics simulations quantitatively reproduced the cooperative effect of multiple mutations on labeling rate.

## Abstract 2

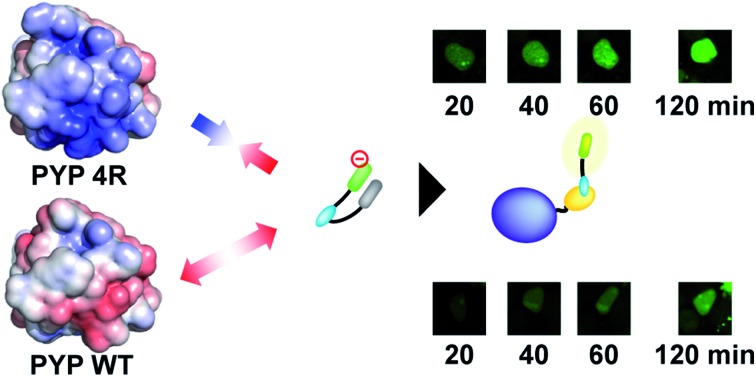
Quick and no-wash labeling of intracellular proteins was achieved in live cells using a PYP-tag mutant and a membrane-permeable fluorogenic probe with modular structures.

## Introduction

Fluorescence imaging of cellular proteins has revolutionized biological research by enabling precise spatiotemporal analysis of protein localization and function in living cells.^1^–^6^ Specific protein labeling by synthetic fluorescent probes and complementary protein tags is an emerging technology that utilizes fluorophores with a broad color palette and enables conditional labeling at specific time points.^7^–^12^ In this technique, a genetically fused protein is constructed with the target protein and a protein tag, which specifically binds to the synthetic probe. In order to be useful for a wide range of applications, such systems must fulfil a number of requirements: probes must be membrane permeable in order to visualize intracellular proteins; non-specific subcellular localization should be minimized; the excitation wavelength of the probe should be adjustable; probes should exhibit a fluorogenic response only when bound to the protein tag to avoid background signals from unbound probes;^13^ the labeling rate should be faster than that of the biological phenomena of interest. To date, few protein labeling techniques meet all of these requirements.^13^–^21^ Here, we describe a protein tag and membrane-permeable fluorogenic probe that exhibits all of the above features and demonstrate its use in live-cell imaging of intracellular proteins in 30 min. This labeling method enabled imaging of an epigenome-related protein in nuclei. Moreover, the proposed system utilizes a platform based on modular design principles, so it should be easily extendable to a wide range of applications.

To track the movements of targeted cellular proteins in real-time, it is highly desirable to develop a fluorogenic or activatable probe that does not require time-consuming procedures to wash out free probes.^14^ A variety of fluorogenic probes have been extensively developed: recent examples are FRET-based probes for SNAP-tag,^14^,^22^ BL-tag,^15^ and eDHFR-tag,^16^ Si-rhodamine probes with a spirocyclization mechanism^17^ coumarin derivatives with a photo-induced electron-transfer (PeT) switch,^18^ and a malachite green dye derivative complexed with fluorogen-activating proteins.^19^ Environmental-sensitive fluorogenic probes were also designed using dimethylaminocoumarin^20^ and benzoxadiazole derivatives.^21^ However, FRET-based and quencher-coupled probes require incubation times of 2 h or more, which significantly diminishes the advantages of omitting the washing step.^14^–^16^ The slow labeling rate originates from their relatively large molecular size, which is crucial in membrane permeation. Moreover, the introduction of a quencher often causes steric hindrance in the ligand binding to the protein tag. In contrast, fluorogenic probes based on environment-sensitive fluorophores achieved a labeling rate of less than 30 min.^17^–^21^ However, these fluorogenic switches possessed fixed dye structures and could not utilize alternative fluorophores, which severely limits their resulting spectral range and use in biological systems.

**Fig. 1:**
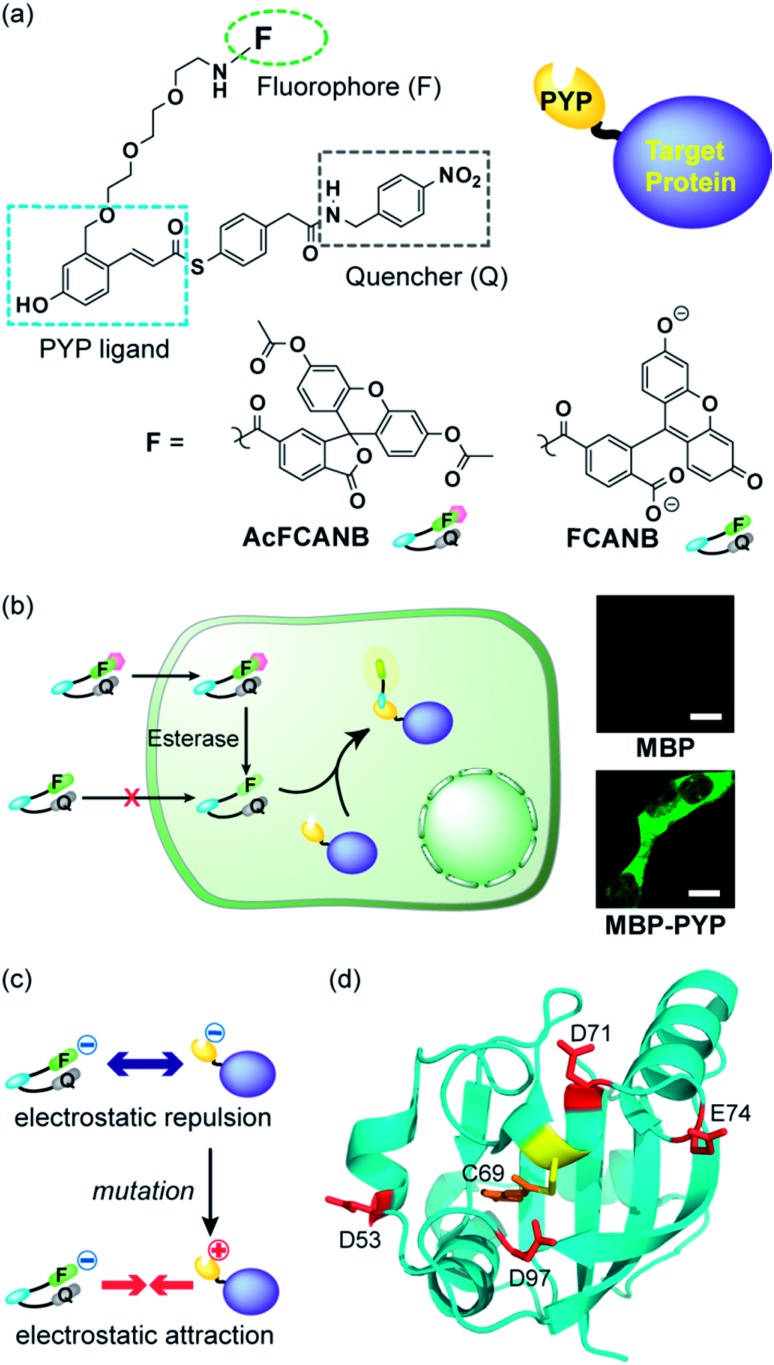
(a) Molecular structures of the fluorogenic probes FCANB and AcFCANB. (b) No-wash live cell imaging of protein labeling with AcFCANB and MBP (top) and MBP–PYP (bottom) expressed in HEK 293T cells. Scale bar: 10 μm. (c) Schematic illustration for the mechanism of labeling acceleration. (d) Structure of PYP showing the reactive Cys-69 surrounded by the four acidic residues targeted for mutation.

We previously developed protein-labeling systems using the Photoactive Yellow Protein (PYP) as a protein tag in combination with either the modular fluorogenic probe FCANB (Fig. 1) or a fixed dye coumarin-based probe.^20^,^23^ The PYP-tag is a small-sized (125 a.a.) water-soluble bacterial protein.^24^,^25^ It forms a covalent bond between thioester derivatives of cinnamic acid or coumarin *via* transthioesterification with Cys-69.^20^,^23^,^26^ FCANB has a triblock modular structure: hydroxy cinnamic acid acts as the PYP ligand, fluorescein the fluorophore, and nitrobenzene the quencher moiety. Nitrobenzene is known to quench fluorophores either by ground-state complex formation or by a PeT process.^27^,^28^ Upon reaction with the PYP-tag, the quencher is eliminated and FCANB recovers its fluorescence. It should be noted that multiple fluorophores could be quenched with this nitrobenzyl quencher.^28^ Thus the FCANB platform allows a variety of fluorophores to be utilized with a wide spectral range, from ultraviolet to near infrared. Since the probe lacks membrane permeability, intracellular protein imaging with FCANB was not possible. In addition, the labeling rate of FCANB and PYP, which is on the order of an hour, is not sufficiently fast.

It is well established that charged residues in or near a protein binding site can affect the rate of binding by polar or charged ligands.^29^ We introduced site-selective mutations on the PYP-tag to modulate surface charges and enhance the labeling rate. We also developed a simple computational model that quantitatively reproduces the cooperative effect of PYP tag mutations on the kinetics of probe binding, which enhances our ability to design new labeling platforms. The strategic design of a biologically applicable fluorogenic protein tag system, supported by a quantitative computational model of the labeling reaction represents a new paradigm for imaging intracellular proteins.

## Results and discussion

### No-wash imaging of PYP with a membrane permeable probe

It has been reported that non-fluorescent fluorescein esters perform as fluorogenic switches enabling analysis of cellular hydrolytic enzyme activities.^30^ Moreover, the fluorescein esters are membrane permeable whereas digested fluorescein molecules are non-permeable and accumulate inside cells.^31^ Based on these observations, a re-designed fluorogenic probe, AcFCANB, with neutral charge was prepared by selective acetylation of FCANB at two hydroxy groups (Fig. 1a). Once incorporated inside the cells, AcFCANB is rapidly digested by cellular innate esterases recovering the anionic FCANB (Fig. 1b).

First, specific labeling of a PYP-fusion protein with the new probe in live cells was evaluated. For the labeling of intracellular proteins, maltose-binding protein (MBP) was selected, and was fused to the N-terminus of PYP-tag (MBP–PYP). After the cells were incubated with AcFCANB for 1 h, fluorescence images of the cells were collected (Fig. 1b). Bright fluorescence was observed inside of cells expressing MBP–PYP, whereas cells expressing MBP alone remained non-stained (Fig. 1b). These results demonstrate that the probe crossed the cell membrane, underwent proper digestion to recover its anionic form, and specifically labeled intracellular PYP-tagged proteins, as desired.

### Design of PYP mutants for accelerating labeling reactions

Next, to improve the labeling rate, we focused on the surface charges of the PYP-tag. In our previous results with coumarin-based probes, a cationic probe could label the PYP-tag more than 30 times faster than an anionic coumarin-based probe, which has a labeling rate comparable to that of FCANB.^20^ These results are consistent with the properties of the PYP-tag, which is also anionic with a pI of 4.3, and has several acidic amino acid residues on the same face as the ligand-binding site (Cys-69). We hypothesized that electrostatic repulsion between the anionic probe (FCANB) and the anionic PYP-tag surface might hinder efficient binding (Fig. 1c). Based on the structure of PYP, three aspartic acid residues and one glutamic acid residue, which are solvent-exposed and on the same face as Cys-69, were identified: D53, D71, E74, D97 (Fig. 1d). We speculated that charge reversal at these residues would reduce repulsive forces, and facilitate interaction between FCANB and PYP-tags, resulting in acceleration of the labeling reaction rate (Fig. 1c). To this end, a series of cationic PYP mutants were designed and created by point mutation of the acidic amino acids: D53R, D71R, E74R, D97R. The distance between the reactive Cys-69 residue and each of the mutated amino acids is summarized in Table 1.

**Table 1:** Kinetic properties of PYP-tag-probes with the distance from reaction center (C69)

PYP	DC_69_ (Å)	*t*_1/2_ ^*a*^ (min)	*k*_2_ ^*a*^ (M^–1^ s^–1^)
WT	—	27	9.8 × 10
D71R	5.4	17	1.2 × 10^2^
D97R	7.0	9.8	2.3 × 10^2^
E74R	10.2	17	1.4 × 10^2^
D53R	18.2	12	2.0 × 10^2^
4R	—	7.1	3.2 × 10^2^

^*a*^All data were obtained in triplicate experiments.

### *In vitro* labeling reactions using PYP mutants

**Fig. 2:**
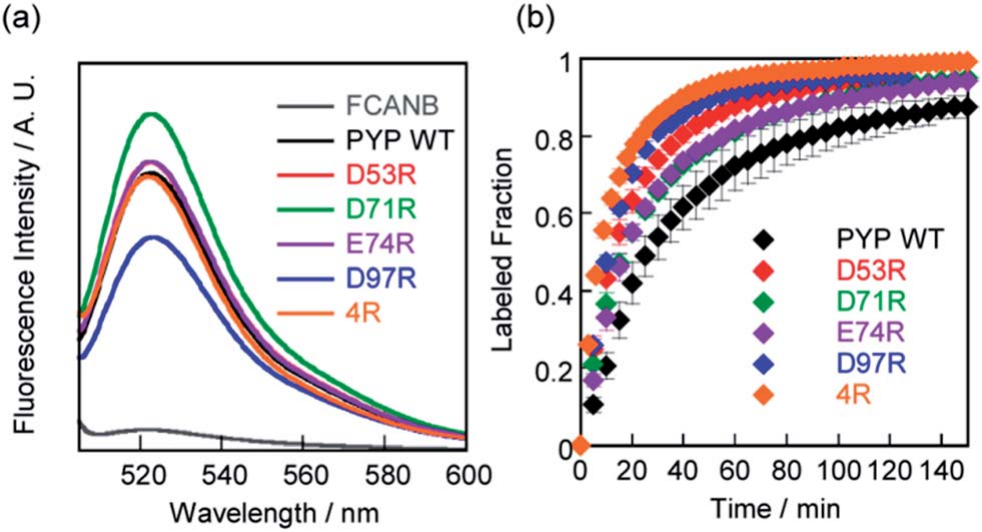
(a) Fluorescent spectra of FCANB reacted with/without PYP or cationic PYP mutants. [Probe]/[PYP] = 2 μM/3 μM. (b) Time course of fluorescence intensity of FCANB at 522 nm with PYP-tags. [Probe]/[PYP] = 6 μM/5 μM. All the measurements were conducted at 37 °C using assay buffer of pH 7.4 including 20 mM HEPES and 150 mM NaCl.

SDS-PAGE analysis confirmed covalent binding between FCANB and each of the mutants (Fig. S3†).^23^Fig. 2a and Table 2 show fluorogenic reaction between FCANB and PYP WT/mutant tags, while the fluorescence spectrum of FCANB alone remained quenched. These results indicate that all of the PYP mutants reacted with FCANB to trigger a fluorogenic response. Fig. 2b shows the time course measurement of fluorescent intensity of FCANB in the presence and absence of PYP tags. All the mutants showed improved binding rates compared with that of the PYP WT (Fig. 2b). The labeling kinetics of each PYP mutant was quantified by the second-order kinetic constant (*k*_2_) and the time required to reach 50% labeling (*t*_1/2_) (Table 1).

**Table 2:** Fluorogenic properties of FCANB before and after the reaction with PYP proteins

PYP	None	WT	D53R	D71R	E74R	D97R	4R
Fold activation^*a*^ (522 nm)	1	14	14	17	15	11	14

^*a*^Fold-activation values were calculated based on the peak fluorescence intensities of FCANB at 522 nm.

The contribution to the labeling rate enhancement varied considerably among the mutants. While the *k*_2_ for PYP WT was 98 M^–1^ s^–1^, the D71R and E74R mutants showed similar improvement in the kinetics (*k*_2_ = 120 and 140 M^–1^ s^–1^). The fastest labeling rate was observed for the D97R mutant with a *k*_2_ of 230 M^–1^ s^–1^, followed by D53R with a *k*_2_ of 200 M^–1^ s^–1^ (Table 1, Fig. 2b). The change in the labeling rate of the mutants is not a simple function of the distance between the mutated residue and Cys-69. For example, the mutation D53R had a significantly greater contribution to the labeling kinetics than either D71R or E74R, which are located much closer to Cys-69 (Table 1). We attribute this lack of correlation between proximity and rate to the relatively large size of the probe compared with that of the binding site. It should be noted that the mutations also affect the brightness of the fluorophore (Fig. 2a). For example, D97R exhibited the highest labeling rate among four mutants, but a significant loss of fluorescence was also observed. Specific adhesion of the fluorophore to the protein surface could be one reason for the partial quenching of the fluorescent molecules. The fluorescein moiety of the probe protrudes from the binding pocket but is located close to the protein surface. Therefore, local interactions between the probe and charged amino acids of the mutants may not be negligible. On the other hand, other cationic mutants afforded comparable or even higher fluorescent intensities than WT (see D71R in Fig. 2a). Thus appropriate interactions between the protein surface and the fluorophore might cancel local adhesion of the fluorophore, preventing undesired fluorescence quenching. These results prompted us to develop multiple mutants to induce cooperative effects on labeling kinetics and fluorescence enhancement. PYP 4R was designed by mutating all four targeted acidic amino acids to arginine. PYP 4R showed the highest labeling rate, as expected, with a *k*_2_ of 320 M^–1^ s^–1^. Moreover, a fluorescent intensity similar to that of the WT was fully recovered (Fig. 2a).

### Quantitative model of the labeling reaction

**Fig. 3:**
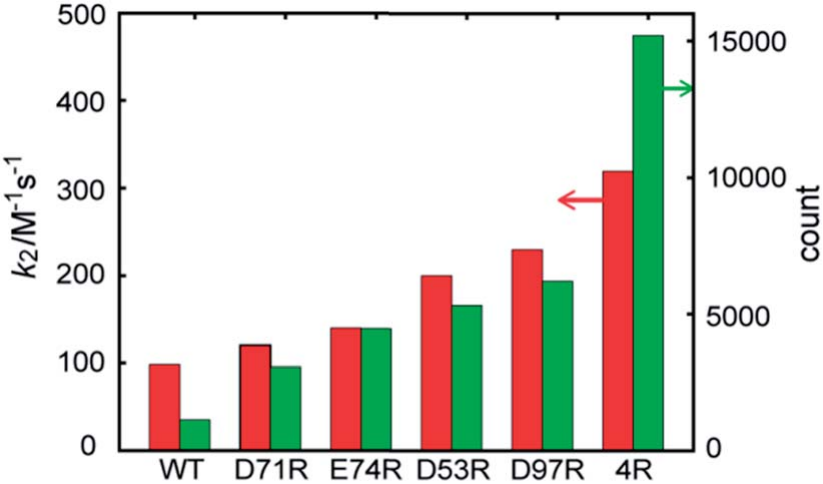
FCANB binding density resulting from the implicit solvent MD simulations (green bars, right axis) and the experimentally observed labeling rates (red bars, left axis).

The contribution of each mutation to the labelling rate did not correlate inversely with the distance between the targeted residue and the reactive Cys-69, as predicted by simple proximity-based models.^32^ These results prompted us to directly model the effect of each mutant on the labeling rate by MD calculations. In order to gain insight into the effects of the mutations on long-range FCANB–PYP-tag interactions, we modeled the system as follows: we initialized the probe in the bulk region at 30 different starting positions. In each initial configuration, we randomly placed the probe on the surface of a sphere of radius 50 Å, centered on the geometrical center of the PYP-tag. A single 100 ns implicit solvent simulation was run for each of the 30 configurations and for each of the 6 PYP-tag constructs. The binding propensity was characterized using six reference atoms on the probe (Fig. S6†). We compared the distance between the geometric center of the reference atoms in each snapshot to those in the bound state. Fig. 3 demonstrates that the density of snapshots within a threshold distance of 6 Å and *k*_2_ correlate well, even for the D53R mutant that does not follow the proximity rule.

Moreover, the MD simulations reveal why the proximity rule does not work for large-sized ligands like FCANB. First we confirmed that the conformational ensemble of “bound” conformations was not significantly affected by use of implicit solvent (see ESI† “Explicit water simulations”), which implied that the highly efficient GPU-accelerated MD calculations used here were sufficient to recapitulate the binding rates of large charged ligands on charged proteins. Next, we investigated the distribution of contacts between the FCANB ligand and each of the PYP constructs (see ESI† “Distribution of probe-tag contacts”). This analysis supported a scenario wherein the position, not only the proximity, of the charges is important for proper binding. These results, in turn, suggest that long-range interactions between FCANB and PYP-tags, which can steer the orientation of the probe, have a significant effect on the labeling rate. We note that the effect each mutation on the p*K*_a_ of cysteine was computed as well, but even the mutation with the largest predicted effect (D97R) was very small (<1% change in the population of oxidized cysteine). We also carried out MD simulations under the exact same conditions using a positively charged ligand (RGT) and found that the trend in density was reversed, as expected, confirming the sensitivity of the solvent model (see ESI “Implicit solvent MD simulation of RGT”, Fig. S11†).

### Live-cell imaging of PYP mutant fusion proteins

We further confirmed that no-wash imaging of intracellular proteins was enhanced using the PYP 4R mutant expressed with maltose-binding protein (MBP) or actin fused to blue fluorescent protein (BFP) in HEK293T cells (MBP–PYP, PYP–BFP–actin and MBP–PYP–NLS) (Fig. S12 and S13†). The bright fluorescence signal was only detected from inside the cells for MBP–PYP 4R, similar to MBP–PYP WT (Fig. S12†). Nontransfected cells or cells expressing MBP remained non-stained (Fig. S12†). Actin was also imaged by using PYP 4R fused to BFP. Colocalization of PYP 4R with BFP was clearly observed (Fig. S13†). MBP–PYP WT–NLS and MBP–PYP 4R–NLS exhibited fluorescence from nuclei with comparable intensities (Fig. S12†). These results indicated that the cationic mutation of the PYP-tag did not cause non-specific accumulation or aggregation of fusion proteins.

**Fig. 4:**
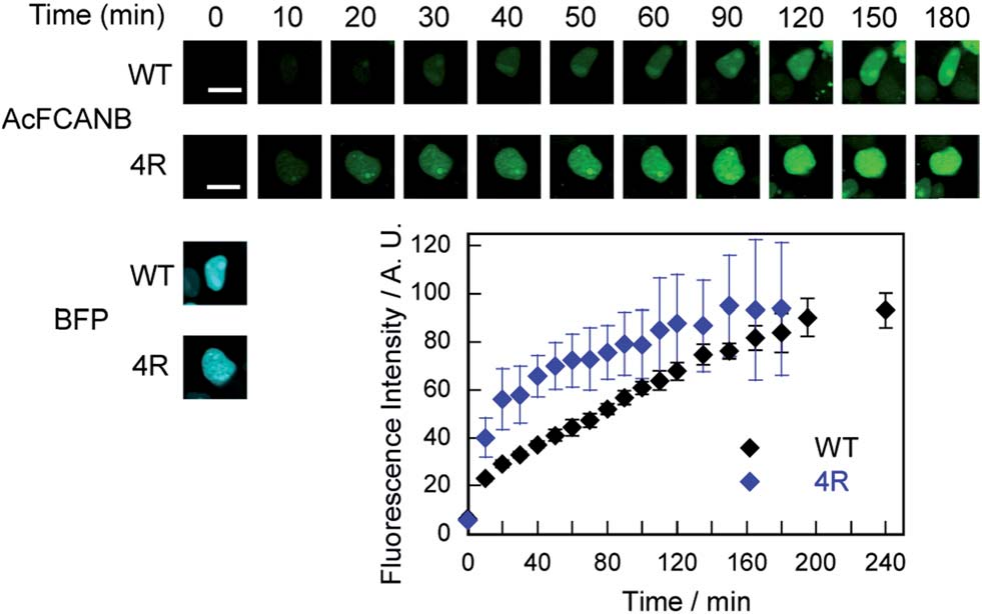
(top) Time-lapse imaging of PYP WT–BFP–NLS and PYP 4R–BFP–NLS expressed in HEK 293T cells with AcFCANB. The images were collected every 10 min after the addition of the probe (2 μM), with the excitation at 473 nm by using a 490–590 nm emission filter for AcFCANB, and with the excitation at 405 nm by using a 420–520 nm emission filter for BFP. (bottom) Labeled fraction of PYP against incubation time (*N* = 3). The quantification was conducted by selecting cells exhibiting the equivalent level of fluorescence of BFP at *T* = 0 min. Scale bar: 10 μm.

Time-lapse imaging showed that PYP 4R accelerated labeling reactions compared with PYP WT (Fig. 4). PYP WT and PYP 4R were fused with BFP and NLS (PYP–BFP–NLS) and were expressed in nuclei. For the quantification of the fluorescence signals, BFP was used to select cells that express the PYP proteins in an equivalent level. After the addition of AcFCANB to the cells, detectable fluorescence appeared in the nuclei with PYP 4R in 10 min. PYP 4R–BFP–NLS showed *t*_1/2_ of 20 min, whereas PYP-WT needed more than 1 h to reach *t*_1/2_. The labeling time required to visualize PYP-tag-fused protein was significantly shortened in live cells. These results are consistent with both *in vitro* measurements and MD simulations of PYP 4R showing improved labeling rates over PYP WT owing to electrostatic interactions. The protein labeling kinetics in live cells seemed to be slower than *in vitro* kinetics. Considering the fact that the deacetylation of diacetylfluorescein by endogenous esterases is sufficiently fast,^31^ one probable reason for the difference between live-cell and *in vitro* experiments is that the penetration rate of the probe through plasma membrane was relatively slow and affected the imaging kinetics of PYP proteins in live cells. Taken together, the AcFCANB/PYP 4R-tag achieves no-wash imaging of intracellular proteins by a membrane-permeable fluorogenic probe with a modular platform allowing versatile fluorophores within a feasible working time.

### Live-cell imaging of heterochromatin protein 1α in nuclei

Finally, intracellular tracking of heterochromatin protein 1α was conducted to demonstrate the biological feasibility of a AcFCANB/PYP mutant tag. HP1 is a key player in the epigenetic control of gene expression by forming and maintaining heterochromatin structures. A recent study revealed that HP1 recognizes a methylated lysine residue at histone H3;^33^ however, the local dynamics of HP1 in living cells has not been fully elucidated.

**Fig. 5:**
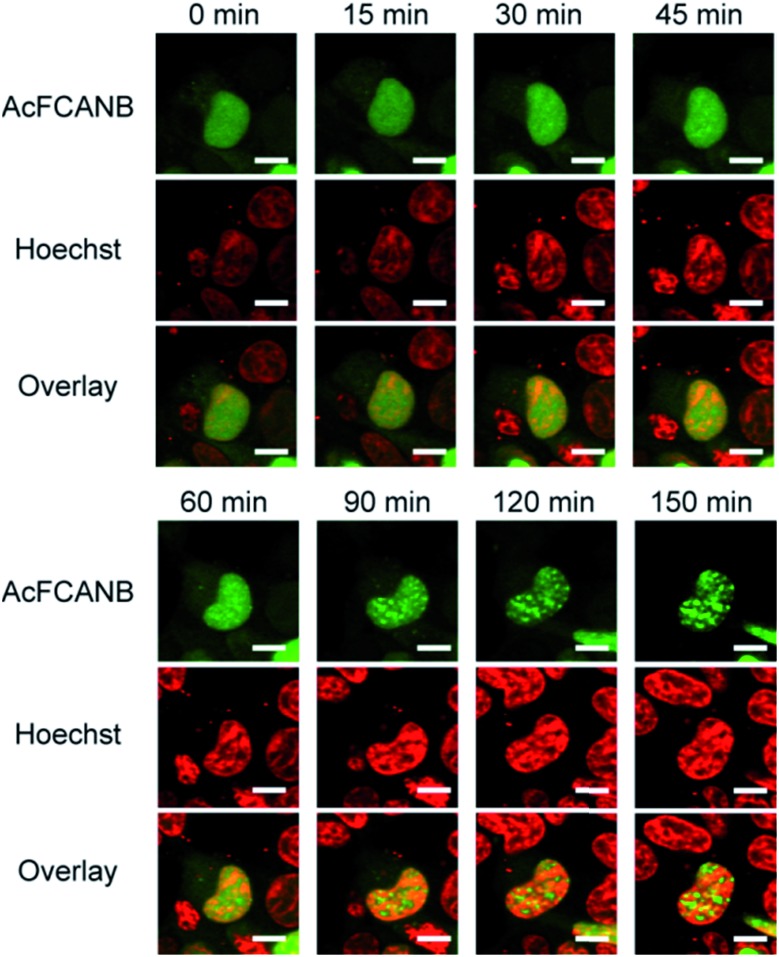
Live-cell imaging of localization of PYP 4R–HP1α expressed in HEK 293T cells co-stained with AcFCANB and Hoechst 33342. The confocal fluorescent images were collected with the excitation at 473 nm for AcFCANB and at 405 nm for Hoechst. Scale bar: 10 μm.

We performed time-lapse imaging of PYP 4R-fused HP1α expressed in HEK293T cells (Fig. 5). At first sight, HP1α is stained uniformly in the nuclei, then the fluorescence signals are gradually concentrated in discrete spots.^34^,^35^ WST assays confirmed that the effect of phototoxicity was negligible under the current experimental conditions (Fig. S15†). The results indicate that a dynamic epigenomic event occurs within a few hours and is successfully captured by using the current probe-tag pair.

## Conclusions

In conclusion, we developed a fluorogenic tool for labeling intracellular proteins by engineering the PYP-tag and using a membrane-permeable probe with a modular design. Labeling was effectively accelerated by selective cationic mutations of the PYP-tag to control electrostatic interactions between the anionic probe and PYP. The resulting AcFCANB/PYP 4R pair enabled no-wash imaging of intracellular proteins in a desirable time frame (*t*_1/2_ < 30 min), without accumulation or adhesion of the tag protein or the probe to non-targeted organelles. Another prominent feature of this work is that *in vitro*, *in silico*, and live-cell results were highly consistent, and enabled the physical basis of the improved reaction rate to be clarified. These results will enable future improvements in the design of novel probe-tag pairs. Although the modular design approach used here resulted in a relatively large probe scaffold, it has the advantage of meeting multiple requirements that are lacking in current labeling systems. In particular, the combination of membrane-permeability, fluorogenicity, and optimized labeling kinetics enables imaging of various biological phenomena to be elucidated, including the epigenetics study of HP1 shown here.

## Experimental section

### Fluorescence spectroscopy

The fluorescence spectra were recorded after the labeling reaction was completed. FCANB (2 μM) was reacted with or without PYP-tag (3 μM) in assay buffer (pH 7.4 20 mM HEPES containing 150 mM NaCl) at 37 °C overnight. The fluorescence spectra were recorded at an excitation wavelength of 501 nm with a slit width of 2.5 nm for both excitation and emission.

### Kinetic analyses of protein labeling reactions

The time required for labeling half of the PYP-tags (*t*_1/2_) and the second-order rate constant for the labeling reaction between the probe (FCANB) and the proteins were determined using previously reported procedures.^20^ To estimate *t*_1/2_, the fluorescence intensity at 522 nm was measured at an excitation wavelength of 501 nm with a slit width of 2.5 nm. For estimation of *k*_2_, the fluorescence intensity at 522 nm was monitored at an excitation wavelength of 496 nm and a slit width of 5.0 nm.

### Intracellular fluorescence imaging of MBP, MBP–PYP, MBP–PYP–NLS, PYP–BFP–actin and PYP–BFP–NLS

HEK 293T cells were transfected with pcDNA3.1(+)–MBP, pcDNA3.1(+)–MBP–PYP WT, pcDNA3.1(+)–MBP–PYP 4R, pcDNA3.1(+)–MBP–PYP WT–NLS, and pcDNA3.1(+)–MBP–PYP 4R–NLS by using Lipofectamine 2000 (Invitrogen) as the lipofection reagent, according to the manufacturer's protocol. Lipofectamine 3000 (Invitrogen) was used for transfection of pcDNA3.1(+)–PYP 4R–BFP–actin, pcDNA3.1(+)–PYP WT–BFP–NLS and pcDNA3.1(+)–PYP 4R–BFP–NLS. Nontransfected cells (mock) were also prepared without plasmids. Following incubation at 37 °C for 24 h, the cells were washed 3 times with HBSS. The cells were then incubated with AcFCANB (500 nM to 5 μM) in DMEM for 30 min (PYP–BFP–actin) or for 60 min (MBP–PYP, MPB–PYP–NLS). Confocal laser scanning microscopy images of the cells were obtained with excitation at 473 nm. In the time-lapse imaging experiments, fluorescence images of PYP–BFP–NLS-expressing cells were collected every 10 min after the addition of the probes to the culture medium. Average fluorescence intensity values (*n* = 3) were calculated and plotted against time.

### Live-cell imaging of HA–PYP 4R–HP1α

HEK293T cells were transfected with pcDNA3.1(+)–HA–PYP 4R–HP1α, pcDNA3.1(+)–HA–PYP WT–HP1α, and pcDNA3.1(+) (mock) using Lipofectamine 3000 (Invitrogen), following the manufacturer's protocol. After incubation at 37 °C for 24 h, the cells were washed 3 times with HBSS, and 2 μM AcFCANB in DMEM was added, followed by further incubation for 60 min. The cells were transferred to DMEM containing 10% FBS and costained with Hoechst 33342 (250 ng ml^–1^). Fluorescence images of the cells were obtained with excitation at 473 nm for AcFCANB and 405 nm for Hoechst 33342.

### Forcefield development of FCANB

The full-length structure of FCANB was reconstructed using MarvinSketch 6.1.3, and then the structure was submitted to Antechamber in AmberTools 13 for forcefield development. In Antechamber, the AM1-BCC charge method was selected.

### *In silico* mutant development

The OSCAR-star side-chain modeling method^36^ with default settings was used to generate all mutant structures of PYP used for computational simulations.

### Molecular dynamics of PYP–FCANB binding in implicit solvent

The 30 FCANB structures were randomly initialized, using full-length probes of 5 different internal molecular conformations, onto the surface of a sphere with a radius 50 Å centered on the geometrical center of PYP. We prepared topology files by t leap in AmberTools 13. Here, we set up a sphere of radius 80 Å wherein FCANB could move freely, and distance restraints forced the ligand back into the sphere if FCANB escaped. The AMBER99SB forcefield^37^ was used for PYP, using a modified generalized Born parameter set denoted as model II in Onufriev *et al.*^38^ The solvation term was not included in this simulation. The PMEMD tool in AMBER 12 was used for energy minimization and molecular dynamics simulations. No periodic boundary was used, and the cutoff length was set to 9999 Å. The simulation protocol was as follows. First, 500 and 5000 steps of energy minimization using the steepest descent method were performed using the CPU-only and GPU-accelerated versions of PMEMD, respectively. Here, we applied positional restraints of 1 kcal mol^–1^ to all heavy atoms. Then, we ran a 100 ns MD simulation at 300 K controlled by the Andersen thermostat.^39^ The SHAKE^40^ algorithm was used to constrain distances between heavy atoms and bonded hydrogen atoms, and the timestep was set to 2 fs. The MD coordinates were stored every 10 ps.

## Supplementary Material

Supplementary information
