# Supplementary material for: Design of a protein tag and fluorogenic probe with modular structure for live-cell imaging of intracellular proteins
Source: Chem Sci. 2015 Sep 30;7(1):308–14. doi: 10.1039/c5sc02351c (PMC5952543; doi:10.1039/c5sc02351c)
Supplement: Supplementary file 1 [file SC-007-C5SC02351C-s001.pdf]

Electronic Supplementary Material (ESI) for Chemical Science.
This journal is © The Royal Society of Chemistry 2015

Supporting Information

Design of a Protein Tag and Fluorogenic Probe with Modular Structure for Live-Cell Imaging of Intracellular Proteins

Authors: Yuko Kamikawa,[b] Yuichiro Hori,[a],[b],[c] Kazuo Yamashita,[b] Lin Jin,[b] Shinya Hirayama,[a] Daron M. Standley[b] and Kazuya Kikuchi*[a],[b]

[a] Graduate School of Engineering, Osaka University, Suita, Osaka 565-0871, Japan

[b] Immunology Frontier Research Center, Osaka University, Suita, Osaka 565-0871, Japan

[c] JST, PRESTO, Suita, Osaka 565-0871, Japan

*Correspondence author: kkikuchi@mls.eng.osaka-u.ac.jp

Table of Contents

1. Supporting methods 2
  - Materials and instruments 2
  - Syntheses of compounds 2
  - Preparation of plasmids 4
  - Preparation of recombinant proteins 7
  - Protein labeling reactions visualized by SDS-PAGE 7
  - UV-Vis spectra of FCANB and PYP WT 7
  - Immunofluorescence 7
  - WST assay 8
  - Truncated FCANB 8
  - Molecular dynamics of PYP-tFCANB binding in explicit solvent 9
  - pKa calculations 9

2. Supplementary Text 10
  - Explicit water simulations 10
  - Distribution of probe-tag contacts 11
  - Implicit solvent MD simulation of RGT 12

3. Supplementary Figures 13

1. Supporting methods

Materials and instruments

General chemicals were of the best grade available and supplied by Tokyo Chemical Industries, Wako Pure Chemical Industries, Sigma-Aldrich Chemical Co, and Kishida Chemical Co, and were used without further purification. Enzymes for subcloning experiments were purchased from Takara Bio and New England Biolabs. Oligonucleotides were purchased from Greiner Bio-One or Life technologies. The plasmid pTag-BFP-actin was purchased from Evrogen.

NMR spectra were recorded on a Bruker AVANCE III HD 500 instrument at 500MHz for ^{1}H and 125 MHz for ^{13}C NMR. High-resolution mass spectra (HRMS) were recorded on a JEOL JMS-700. ESI-TOF MS was recorded on a Waters LCT-Premier XE. Silica gel column chromatography was performed using BW-300 (Fuji Silysia Chemical Ltd.). High-performance liquid chromatography (HPLC) purification was performed with an Inertsil ODS-3 column (4.6 mm or 10.0 mm × 250 mm; GL-Science, Inc.). Fluorescence spectra were measured using a Hitachi F7000 spectrometer with a photomultiplier voltage of 700V. UV-vis absorption spectra were obtained using a Shimadzu UV-2450 spectrometer. Fluorescence microscopic analyses were performed using a confocal laser scanning microscope (Olympus, FLUOVIEW FV10i) equipped with a 60× lens.

Syntheses of compounds

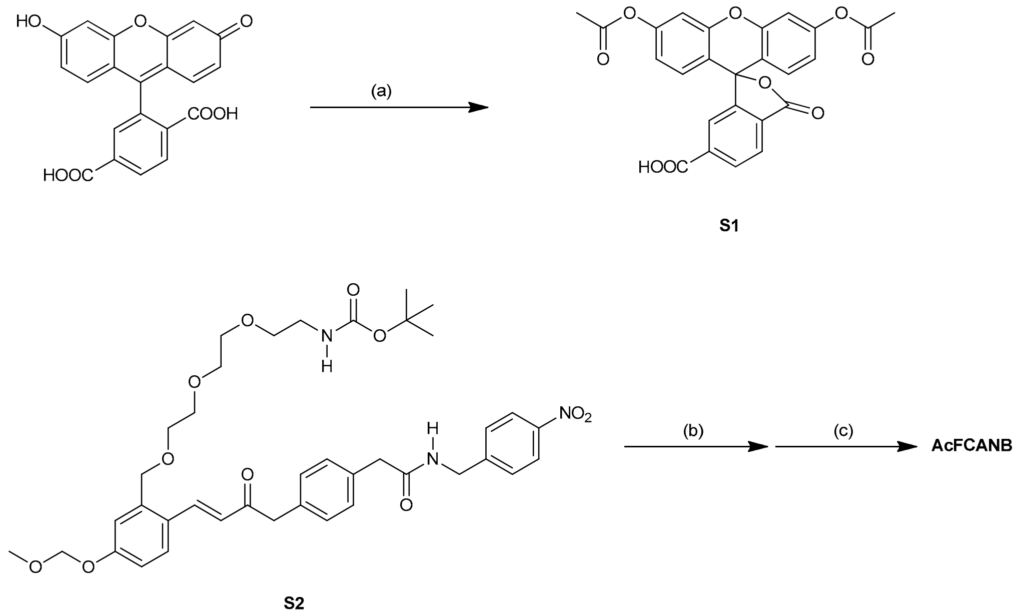

Scheme S1. Synthesis of AcFCANB. (a) Ac_2O, Cs_2CO_3/ DMF (b) TFA/ DCM (c) compound

S1, WSCD·HCl, NHS, TEA/ DMF.

Synthesis of FCANB and compound S2 was reported previously.[1]

<u>AcFCANB</u>

Acetic anhydride (12 mg, 0.12 mmol), 6-carboxyfluorescein (15 mg, 0.03 mmol) and Cs_2CO_3 (54 mg, 0.17 mmol) were dissolved in *N*,*N*-dimethylformamide (DMF, 1 ml), and the reaction mixture was stirred for 5 h at RT. After removing the solvent, the residue was diluted with ethylacetate, washed with 10% citric acid aq., dried over Na_2SO_4 and evaporated to yield compound S1. Compound S1 was used without further purification.

Compound S2 (46 mg, 0.061 mmol) was dissolved in dichloromethane (DCM, 5.0 mL), trifluoroacetic acid (TFA, 1.0 mL) was added, and the mixture was stirred for 30 min at RT. After removing the solvent, deprotected S2-NH_2 was obtained. Compound S1, 1-Ethyl-3-(3-dimethylaminopropyl)carbodiimide hydrochloride (18 mg, 0.09 mmol) and *N*-hydroxysuccinimide (11 mg, 0.09 mmol) were dissolved in DMF (1 ml) and stirred overnight at RT. S2-NH_2 and triethylamine (TEA, 6.2 μl, 0.05 mmol) were added to the reaction mixture and stirred for an additional 4 h at RT. After removing the solvent, the crude residue was purified using silica-gel column chromatography with DCM/methanol (97/3) as an eluent to yield AcFCANB (8.4 mg, y. 22%).

^{1}H-NMR (500MHz, DMSO-*d*$_6$): δ 2.28 (s, 6H, **bb**), 3.42-3.52 (m, 12H, **o, p, q, r, s, t**), 3.57 (s, 2H, **e**), 4.41 (d, 2H, **c**, J_{cd}=5.9 Hz), 4.53 (s, 2H, **n**), 6.76 (dd, 1H, **k**, J_{kj}=8.5 Hz J_{kl}=2.2 Hz), 6.82 (d, 1H, **i**, J_{ih}=15.6 Hz), 6.82 (d, 1H, **l**, J_{lk}=2.2 Hz), 6.93 (m, 4H, **y**, z), 7.30 (s, 2H, **aa**), 7.39 (m, 4H, **f, g**), 7.48 (d, 2H, **b**, J_{ba}=8.6 Hz), 7.76 (d, 1H, **j**, J_{jk}=8.5 Hz), 7.80 (s, 1H, **x**), 7.82 (d, 1H, **h**, J_{hj}=15.6 Hz), 8.13 (d, 1H, **v**, J_{vw}=8.1 Hz), 8.19 (m, 3H, **a**, **w**), 8.74 (t, 1H, **u**, J_{ut}=5.5 Hz), 8.79 (t, 1H, **d**, J_{dc}=5.9 Hz), 10.18 (s, 1H, **m**); ^{13}C-NMR (125MHz, DMSO-*d*$_6$): δ 21.46, 31.26, 42.25, 42.40, 69.08, 69.64, 69.99, 70.12, 70.14, 70.62, 81.68, 111.00, 115.82, 116.19, 116.83, 119.20, 122.06, 122.78, 123.56, 123.99, 125.80, 125.88, 128.03, 128.63, 129.64, 129.87, 130.40, 130.47, 130.71, 134.94, 138.19, 139.03, 140.98, 141.39, 146.90, 148.05, 151.26, 152.63, 160.49, 164.84, 168.26, 169.19, 170.60, 187.55; HRMS (FAB$^+$) *m/z*: calcd for $[M+H]^+$ 1052.2912, found 1052.2910.

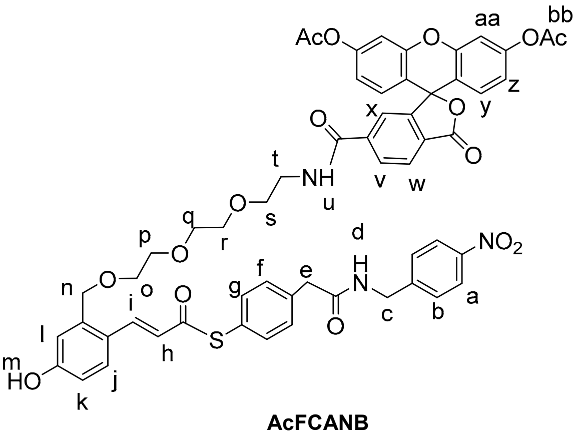

AcFCANB

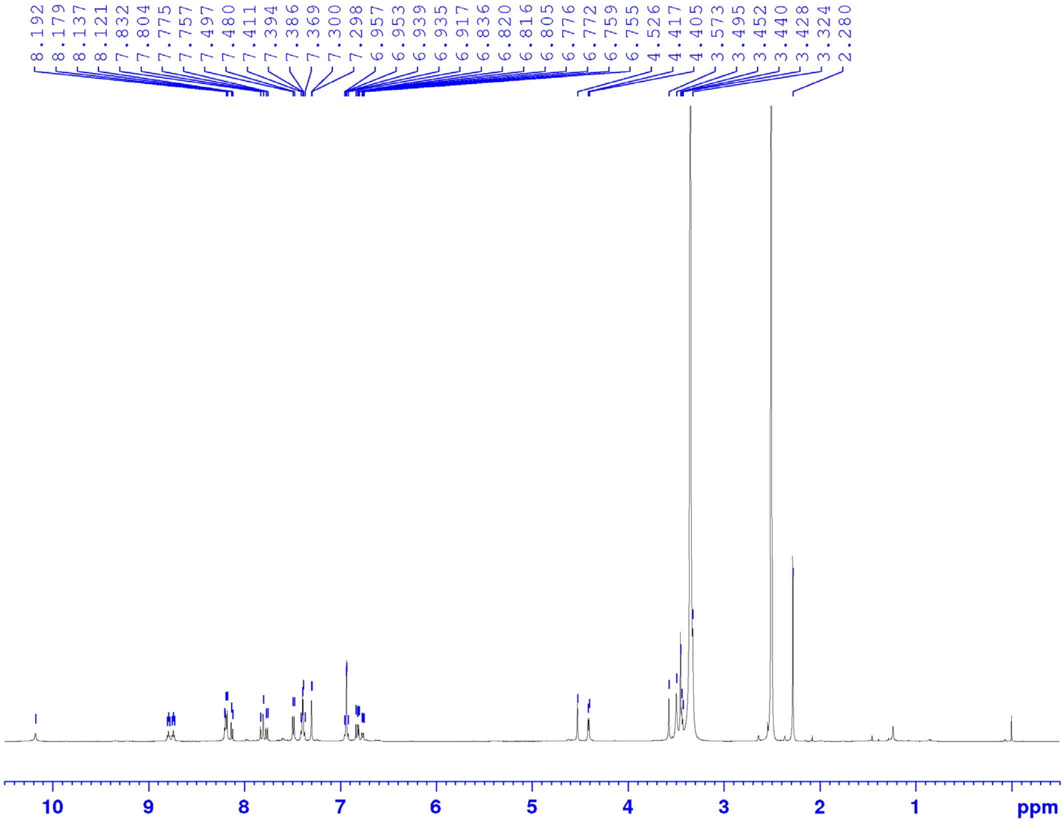

Preparation of plasmids

Plasmids for the single cationic mutants (**pET21b-PYP-D53R**, **PYP-D71R**, **PYP-E74R**, and **PYP-D97R**) were prepared using a QuikChange Mutagenesis Kit, using the primers listed below. CGC was selected as the triplet sequence coding for

arginine. The mutation site is highlighted in red. For construction of **PYP-4R**, **PYP-D71RE74R** was first prepared, a point mutation was induced at D97R, and finally a mutation was induced at D53R, using above-mentioned primers.
PYP-D53R: CATCACCGGCCGC**CGC**CCGAAGCAGGTC
PYP-D71R: GCCCCGTGCACT**CGC**AGCCCGGAGTTC
PYP-E74R: CCGTGCACTGACAGCCCG**CGC**TTCTACGGCAAGTTCAAG
PYP-D97R: GATGTTCGAGTACACCTTC**CGC**TACCAAATGACGCCCACG
PYP-D71RE74R: GCCCCGTGCACT**CGC**AGCCCG**CGC**TTCTACGGCAAG

pcDNA-MBP, pcDNA-MBP-PYP

The plasmids pcDNA-MBP and pcDNA-MBP-PYP were prepared as described previously.[2]

pcDNA-MBP-PYP4R

The PYP4R-tag DNA fragment was prepared from pET21-PYP4R by PCR using primers with sequences GATGACAAGCTTATGGAACACGTAGCCTTC and ACTGCAGAATTC TCAGACGCGCTTGACGA. The fragment was digested with HindIII and EcoRI then ligated to a similarly digested plasmid, pcDNA3.1(+)-MBP, to generate pcDNA-MBP-PYP4R.

pcDNA-MBP-PYP-NLS

The MBP DNA fragment was prepared from pcDNA3.1(+)-MBP-PYP by PCR using primers with the sequences TAGATAGCTAGCGCCACCATGAAAATCGAAGAA GGTAAACT and TAGATCAAGCTTCCTTCCCTCGATCCC. The PYP DNA fragment was similarly prepared from the same pcDNA3.1(+)-MBP-PYP plasmid, using primers with the sequences GATGACAAGCTTATGGAACACGTAGCCTTC and AAGATC GGATCCGACGCGCTTGACGA. The MBP fragment was digested with NheI and HindIII, then ligated to the similarly digested vector pcDNA3.1(+) to form pcDNA3.1(+)-MBP, followed by insertion of the PYP fragment, which had been digested with HindIII and BamHI, to generate pcDNA3.1(+)-MBP-HindIII-PYP. The NLS DNA fragment was prepared from pcDNA3.1(+)-PYP-NLS by PCR using primers with the sequences ACCCGTGGATCCGGAGGATCTGGAGGATCTG and TGGCGC GAATTCTCATACCTTTCTCTTTTTCTTAGGATCAAC. The fragment was digested with BamHI and EcoRI then ligated to the similarly digested plasmid pcDNA3.1(+) -MBP-HindIII-PYP to yield the final plasmid pcDNA3.1(+)-MBP-PYP WT-NLS.

pcDNA3.1(+)-MBP-PYP 4R-NLS was prepared using the vector pcDNA3.1(+)-MBP -PYP WT-NLS and the PYP 4R fragment produced from pcDNA3.1(+)-MBP-PYP 4R using primers with the sequences GATGACAAGCTTATGGAACACGTAGCCTTC and AAGATCGGATCCGACGCGCTTGACGA. The insert and the vector were digested with Hind III and Bam HI then ligated to generate pcDNA3.1(+)-MBP-PYP 4R-NLS.

pcDNA-HA-PYP 4R-HP1α

The plasmid pGEX-HP1a was a gift from Prof. S. Tajima and Dr. I. Suetake at Osaka University. The plasmid pcDNA-HA-PYP-NLS was prepared as reported previously.[2] The HP1α DNA fragment was prepared from pGEX-HP1α by PCR using primers with the sequences GCTGACGAATTCATGGGAAAGAAAACCAAGC and AAGATC CTCGAGTCAGCTCTTTGCTGTTTCTTTCTC. The HP1α fragment was digested with EcoRI and XhoI and then ligated to the similarly digested plasmid pcDNA3.1(+)-HA-PYP WT-NLS to form pcDNA3.1(+)-HA-PYPWT-HP1α.

pcDNA3.1(+)-HA-PYP 4R-HP1α was prepared using the corresponding PYP WT vector and the PYP 4R fragment prepared above by digestion with Hind III and Bam HI followed by ligation.

pcDNA-HA-PYP4R-BFP-actin

The BFP-actin DNA fragment was prepared from pTagBFP-actin by PCR using primers with the sequences GAATATGGATCCGGATCTGGAATGAGCGAGCTGATTAAGG and GGACTCGAATTCCTAGAAGCATTTGCGGTG. The BFP-actin fragment was digested with Bam HI and EcoRI and then ligated to the similarly digested plasmid pcDNA3.1(+)-HA-PYP 4R-NLS to form pcDNA3.1(+)-HA-PYP4R-BFP-actin.

pcDNA-HA-PYP-BFP-NLS

The BFP DNA fragment was prepared from pTagBFP-actin by PCR using primers with the sequences GAATATGGATCCGGATCTGGAATGAGCGAGCTGATTAAGG and CATCTCGGATCCATTAAGCTTGTGCCCCAG. The BFP fragment was digested with Bam HI and then ligated to the similarly digested plasmid pcDNA3.1(+)-HA-PYP WT-NLS to form pcDNA3.1(+)-HA-PYP WT-BFP-NLS or pcDNA3.1(+)-HA-PYP 4R-NLS to form pcDNA3.1(+)-HA-PYP 4R-BFP-NLS.

DNA sequencing of all the constructs confirmed that the genes were correctly inserted in the target plasmids.

Preparation of recombinant proteins (PYP-WT, PYP-D53R, PYP-D71R, PYP-E74R, PYP-D97R, and PYP-4R)

PYP-WT and single cationic mutant proteins were expressed in BL21(DE3) using the above-mentioned plasmids and purified as previously described.[1] His-tags were introduced for purification by Ni-NTA affinity chromatography and were not removed from the final proteins.

A modified procedure was employed for the preparation of **PYP-4R**. pET21b-PYP4R was expressed in BL21(DE3), harvested, and lysed following a previously described procedure.[3] The cell lysate was purified on an affinity column loaded with IMAC resin (Roche, cOmplete His-Tag Purification Resin), following the manufacturer's protocol.

Protein purity and size were assessed by SDS-PAGE. The purified proteins were dissolved in assay buffer (20 mM HEPES, 150 mM NaCl (pH7.4)), flash-frozen with liquid nitrogen, and stored at –80 °C.

Protein labeling reactions visualized by SDS-PAGE

SDS-PAGE analysis confirmed covalent binding between FCANB and each of the mutants (Figure S3). PYP-tag (10 μM) was reacted with FCANB (15 μM) in assay buffer at 37°C for 1 hour. The reaction mixtures were subsequently analyzed by SDS-PAGE. After fluorescence images were obtained using the AE-6935B VISIRAYS (ATTO Corporation), the gel was stained with Coomassie Brilliant Blue.

UV-Vis spectra of FCANB and PYP WT

The absorption spectra of FCANB with or without PYP were determined in assay buffer. FCANB (10 μM) and PYP WT (15 μM) were reacted at 37 °C for 6 h prior to measurement.

Immunofluorescence

HEK293T cells were transfected with pcDNA3.1(+)-MBP or pcDNA-MBP-PYP, pcDNA-MBP-PYP-NLS using Lipofectamine 2000 (Invitrogen) according to the manufacturer's protocol. The transfected and non-transfected cells were fixed with 3.7% paraformaldehyde in PBS for 15 min at 25 °C, followed by 10 min in methanol at –4 °C, and washed three times with PBS. The fixed cells were further incubated in PBS containing 0.2% Triton X for 10 min at 25 °C, for effective membrane permeation. The cells were then incubated in blocking buffer (5% BSA (Aldrich) in PBS) at 25 °C for 60 min. The cells were probed with anti-MBP (Monoclonal, NE BioLabs) and FITC-conjugated goat anti-mouse IgG (Bethyl Laboratories) to detect MBP-PYP or

MBP. Stained cells were imaged using a confocal laser-scanning microscope with excitation at 473 nm.

WST assay

WST assays were performed using Cell Counting Kit-8 from DOJINDO following manufacturer's protocol modified as follows. HEK293T cells (2 x 10^5 cells) were seeded, transfected with pcDNA3.1(+)-HA-PYP4R-HP1α, and incubated for 16 hours. The cells were then incubated with AcFCANB and irradiated every 15 min over a 150-min period. The irradiation was conducted with the same laser power that was used in the imaging experiments. The cells incubated for 150 min with the probes under a dark condition were used as controls. CCK-8 solutions (200 μl / 2 ml culture) were then added to the dishes, and the cells were incubated for another 60 min. The supernatant were collected and analyzed by measuring absorbance at 450 nm to determine cell survival ratio.

Truncated FCANB

We refer to the truncated structure of FCANB lacking the quencher group and the linker region between the quencher and the PYP-ligand group, as tFCANB. We investigated the stable PYP-bound tFCANB conformations by explicit water MD simulation. The forcefield for Cys-69-bound tFCANB was developed by running gaussian09 and R.E.D.-III. We set up two initial conformations of PYP-bound tFCANB. Here, we manually placed tFCANB in the binding pocket at different orientations. We ran three 100 ns MD simulations for each initial conformation. The method used for this MD was identical to that for PYP-tFCANB binding simulation shown below. Two of the different starting orientations converged to a single orientation. Next, we chose MD snapshots from the three trajectories starting from this stable orientation. The snapshots of each MD trajectory were clustered into 2 clusters using single linkage clustering with a 1Å cutoff as implemented in the g_cluster tool in the Gromacs suite. A total of six representative structures were produced in this way from the three trajectories. A reduced tFCANB-Cys bond was prepared by protonating the sulfur atom of Cys 69 and the connecting oxygen atom of tFCANB. We built a forcefield for the reduced tFCANB using Antechamber in AmberTools 13. We used a representative structure from the top-ranked cluster as the input. REDUCE 3.03 was used to add hydrogen atoms to tFCANB before running Antechamber. The AM1-BCC charge method was selected in Antechamber.

Molecular dynamics of PYP-tFCANB binding in explicit solvent

The WT and D97R mutant forms of the six representative structures were used as the starting conformations for the MD simulations in which the tFCANB ligand was in a "bound" conformation. For simulations starting from an "unbound" conformation, we applied random rotations and translations to the tFCANB molecule, retaining those that satisfied the following criteria: less than three heavy atoms had a minimum distance of < 3.5 Å between tFCANB and PYP; the minimum distance from both the Cα atom of Cys-69 and Glu- or Arg-97 to any heavy atom in tFCANB was less than 15 Å. We generated two conformations for each selected structure, and the same relative tFCANB position and orientation were used for both WT and D97R simulations.

The details of the MD trajectories were as follows. We prepared topology files by tleap in AmberTools 13,[4] and transformed these into Gromacs[5] format by acpype.[6] The systems were solvated in a TIP3P explicit water box with a 10 Å edge distance. Sodium or chloride ions were added to neutralize the system as needed. The AMBER99SB forcefield was used for components of the system other than tFCANB. We used Gromacs 4.5.5 for all MD simulations. The particle mesh Ewald (PME) method was used to calculate the electrostatic potential. The cutoff distances for non-bonded interactions and the timestep for MD were set to 10 Å and 2 fs, respectively. The LINCS[7] algorithm was utilized to constrain distances between hydrogens and bound heavy atoms. The Parrinello-Rahman[8] and v-rescale methods were used for pressure and temperature coupling, respectively, if not otherwise specified below. The MD protocol was as follows. First, 5000 steps of energy minimization were performed by the steepest descent method followed by 20ps of MD at 300K to equilibrate waters and ions in the NVT ensemble, while applying 1000 kJ mol^{-1} nm^{-2} isotropic positional restraints to heavy atoms in the protein and the ligand. Then, we applied successive 1000 step energy minimizations while decreasing the weight of the positional restraints, from 1000 to 1 kJ mol^{-1} nm^{-2}. The system was heated from 50 to 300 K during 100 ps of MD at 1 atm without restraints. Finally, a production MD run for 100 ns was performed at 300 K and 1 atm.

pKa calculations

We utilized the PYP crystal structure (PDB identifier 1OTB) and our models of the mutant PYP constructs. The pKa calculations were carried out using PROPKA 3.1 with default settings.[9]

2. Supplementary Text

Explicit water simulations

The agreement between the density of the implicit solvent binding simulations and experimentally observed binding rates was remarkable for several reasons. First, the actual labeling experiment is irreversible, but our simulation was based on cumulative density of ligand positions. Second, we included the leaving group, which presumably causes some steric hindrance to the FCANB-PYP interaction, preventing perfect binding. Third, we treated the water implicitly, which would be expected to result in artifacts in short-range electrostatic and hydrophobic interactions. Addressing the first issue (covalent bonding) is beyond the scope of our MD study, but the remaining two issues can be addressed by carrying out explicit water MD simulations of the FCANB without the leaving group (tFCANB) and comparing these to the implicit solvent results. Here, we initialized the ligand in the bulk at minimum distance of 6-15 Å from the protein surface close to the binding residue Cys-69 (Figure S7) and again ran all 6 PYP constructs (4 point-mutants, 1 quadruple mutant, and WT) for a total of 6x30 25 ns simulations. When we computed the population of snapshots within a 6 Å threshold as above, we obtained a distribution very different from the longer implicit solvent result, which did not correlate well with the experimentally observed labeling rates. The lack of agreement is unsurprising because each MD run was too short to reach equilibrium even for such a small protein-ligand system. In addition, the biased system setup could introduce errors in the observed statistics. However, the mode of interaction between PYP and FCANB would be expected to be more realistic in the explicit water simulation than in implicit solvent. In order to assess if implicit solvent simulations contained artifacts that affected the resulting structures, we carried out the following exercise. First, we assumed that the densities derived from the implicit volume simulations were accurate. Second, we replaced snapshots within the 6 Å threshold from the implicit solvent runs by snapshots within the same threshold from the explicit solvent MD runs. The total number of explicit water snapshots was less, but the proportions for each mutant and WT were maintained. The resulting distribution, by definition, appears as a scaled-down version of Figure 4. We next assessed the binding propensity in more detail as follows. First, we computed the RMSD of the three reference atoms from each bound ligand in the 6-molecule ensemble. For each RMSD value, we computed a binding probability as

$$p = \exp(-\frac{RMSD^2}{W^2}) \quad (1)$$

with $W = 6$ Å. We then computed the average of this value over the 6 members of the bound ensemble. Finally, we summed this average probability over each explicit water snapshots. In spite of the use of explicit-water snapshots and the more rigorous treatment of binding propensity, the resulting distribution looked very similar to that obtained from the implicit solvent simulations (Figure S8). Thus we concluded that the implicit water simulations did not contain severe artifacts that affected the structure of the FCANB-PYP complexes.

Distribution of probe-tag contacts

To further clarify the effects of the mutations PYP, we investigated contacts between PYP and different parts of FCANB. Since we expected that the explicit solvent simulations were more realistic in terms of the mode of binding, we investigated PYP-tFCANB interactions within the 6Å threshold. Here, we divided tFCANB into three parts; fluorophore, PYP ligand, and linker region of above two (Figure S9), and counted the number of contacts of each atom group. We ignored the linker region from this analysis because it shouldn't be so important in electrostatics driven interactions. The profiles and electrostatic field on the solvent accessible surface (SAS) of PYP constructs are shown in Figure S9B-C and S10. In principle, the distributions of residues contacting the fluorophore reflected the electrostatic field because of the partial negative charge of the atom group. Interestingly, the residues of the WT contacting the fluorophore were in a similar location to those of the bound mode, though there are other surface regions that are positively charged. This is consistent with a scenario in which the electrostatic field steers the ligand toward the bound conformation accelerating binding kinetics. Indeed, D53R and D97R, which showed faster labeling rates, exhibited a similar distribution of contacts. The difference between D53R and D97R appeared in the contacting profile of the PYP-ligand group. D97R could more effectively attract this group to Cys-69. This difference can be explained by the balance of the strength of the electrostatic potential between the fluorophore and PYP-ligand interacting regions in bound conformations. The electrostatic field around Arg-53 in D53R might be too positive compared with the region around Cys-69 since there are other basic residues around Arg-53 (e.g. Arg-52). In contrast, the magnitude of these regions in D97R were more modest. Another example that demonstrates the importance of the electrostatic potential is D71R, where both fluorophore and PYP-ligand groups

made contact with the mutated residue many times. In this case, the positive region of electrostatic potential was distributed on the outside of the binding pocket, and this likely made it difficult for FCANB to enter the binding pocket. In other words, the PYP-ligand group could not make strong contact with Cys-69 due to its attraction to Arg-71. E74R had few direct contacts between Arg-74 and FCANB, thus, we speculate that the main contribution to the acceleration above that of the WT was due to the difference in the net charge. The profile of the quadruple mutant (4R) indicated that the effect of mutations on the binding kinetics is not additive but cooperative. For example, Arg-74, which didn't interact well with FCANB as a point mutant, had many contacts with FCANB in the quadruple mutant, possibly due to the influence of the other two mutated residues (Arg-97 and Arg-71), which are close to Arg-74. Given the proximity of these three residues, it's natural that the electrostatic potential around them was changed drastically from that of the WT. Overall, for larger ligand binding, mutated residues should be selected so that the resulting electrostatic field is able to attract the ligand with a conformation similar to that of the bound state.

Implicit solvent MD simulation of RGT

To assess the robustness of the implicit solvent MD simulation, we applied the same protocol used for FCANB to the RGT ligand with a positively charged fluorophore (Fig. S11). Since WT PYP has a net (–6) negative charge, a positively charged ligand has a much greater preference to bind. In the case of the 4R mutant, RGT binding rate was reduced, as expected. However, the difference between WT and 4R binding rates was not as large as in the case of FCANB, suggesting that RGT is not as sensitive to the overall charge as FCANB. This is consistent with the net ligand charge (+1 for RGT and –2 for FCANB). In addition, the distribution of residues on PYP is more suitable for binding RGT than FCANB.

3. Supplementary Figures

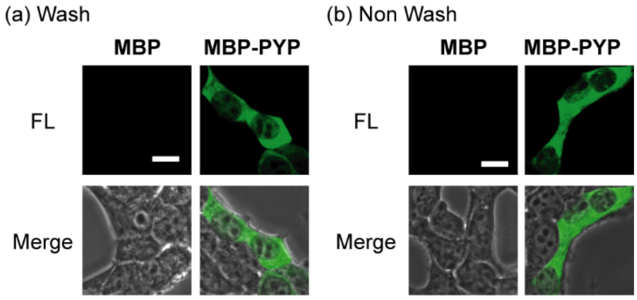

Figure S1. Fluorescent microscopic images of MBP-PYP WT expressing HEK293T cells by AcFCANB with (a) and without (b) washing procedures. Cells expressing MBP only were used as control. AcFCANB was excited at 473 nm and detected at 490-590 nm. Scale bar: 10 μm. Incubation time: 1 hr. [AcFCANB]: 500 nM.

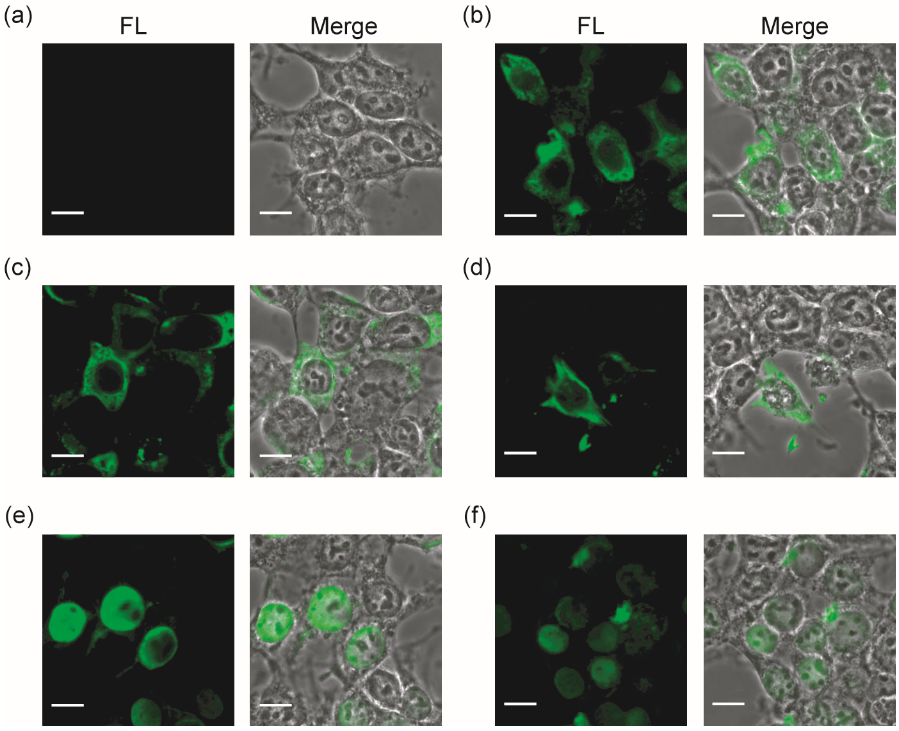

Figure S2. Confirmation of protein expression by Immunostaining. Cells were transfected with mock (Non Transfection) (a), MBP (b), MBP-PYP WT (c), MBP-PYP 4R (d), MBP-PYP WT-NLS (e), MBP-PYP 4R-NLS (f). The cells were probed with anti-MBP. Fluorescence images and their overlays on phase contrast images are displayed in the left and right of each panel, respectively. Scale bar: 10 μm.

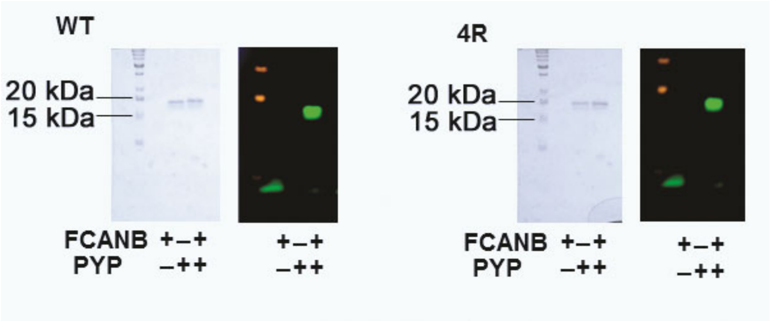

Figure S3. Protein labeling experiments visualized by SDS-PAGE. CBB-stained and fluorescence gel images are displayed on the left and right, respectively. PYP-tag (PYP-WT or PYP-4R, 15 μM) was reacted with FCANB (10 μM).

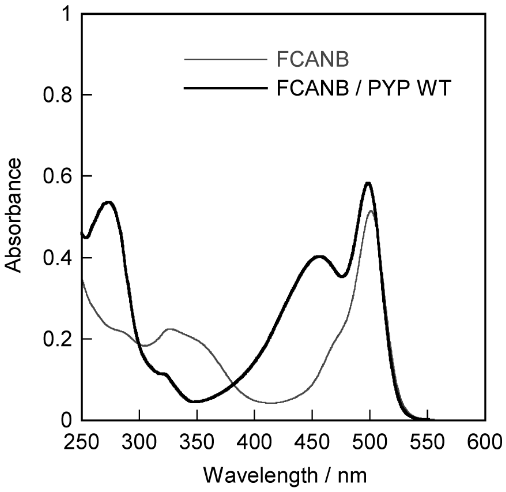

Figure S4. Absorption spectra of FCANB (10 μM) and FCANB / PYP WT (10 μM / 15 μM) in pH 7.4 HEPES buffer.

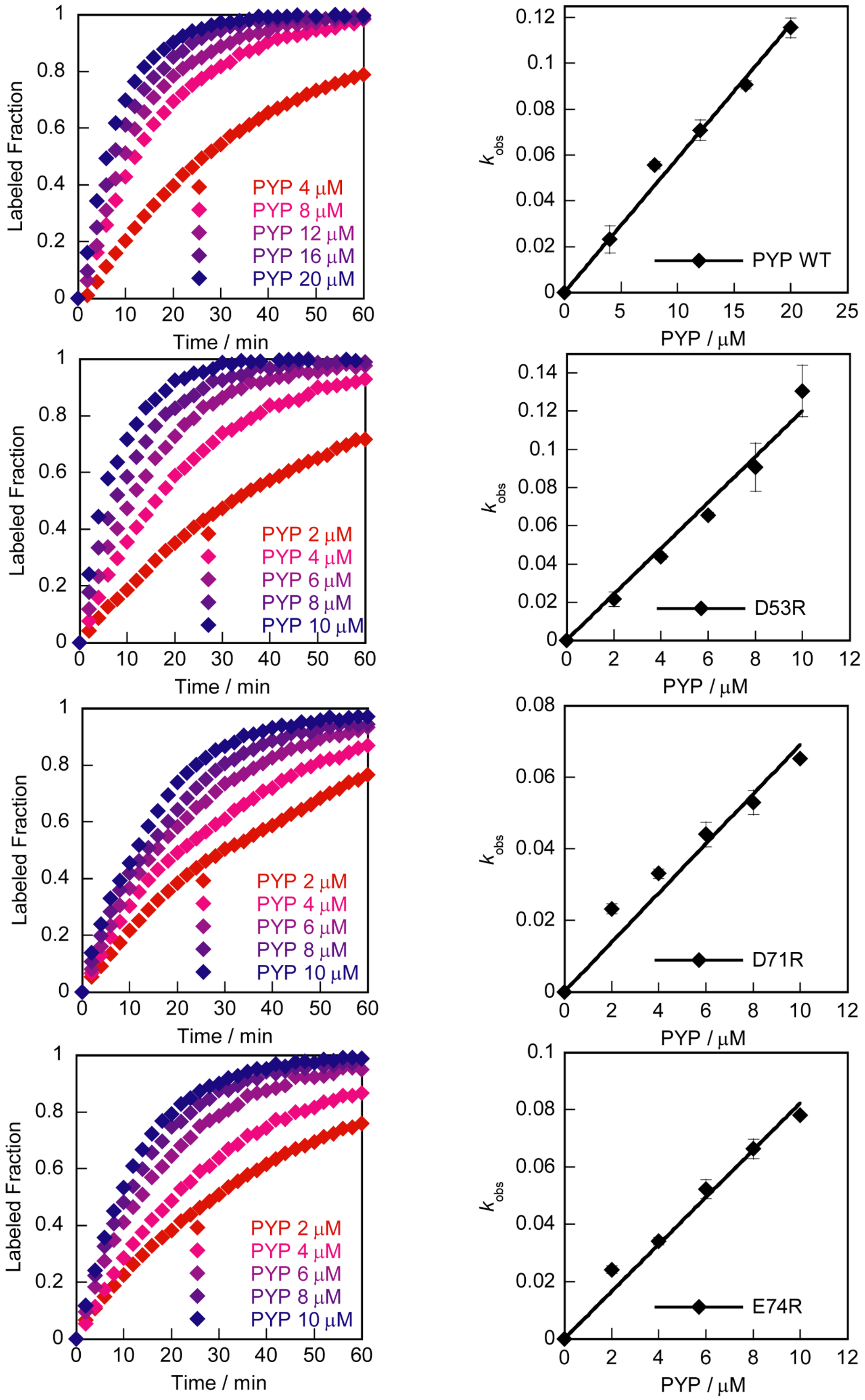
Labeled Fraction
Time / min
PYP 4 μM
PYP 8 μM
PYP 12 μM
PYP 16 μM
PYP 20 μM
k_{obs}
PYP / μM
PYP WT
PYP 2 μM
PYP 4 μM
PYP 6 μM
PYP 8 μM
PYP 10 μM
D53R
D71R
E74R

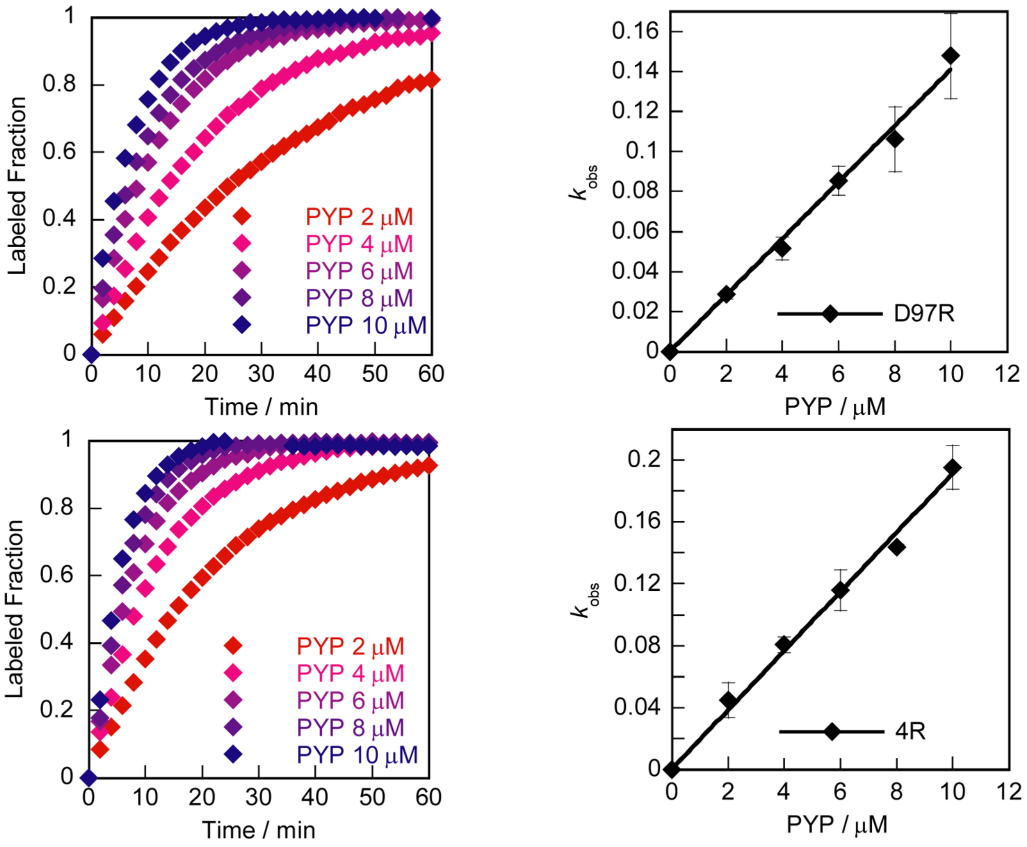

Figure S5. Kinetic analyses of protein labeling reactions with FCANB and PYP. (Left) Reactions of probes (FCANB: 200 nM for PYP WT, 100 nM for PYP mutants) with PYP-tag at various concentrations. (Right) Plots of protein concentration against the pseudo-first-order rate constant of FCANB / PYP binding reactions.

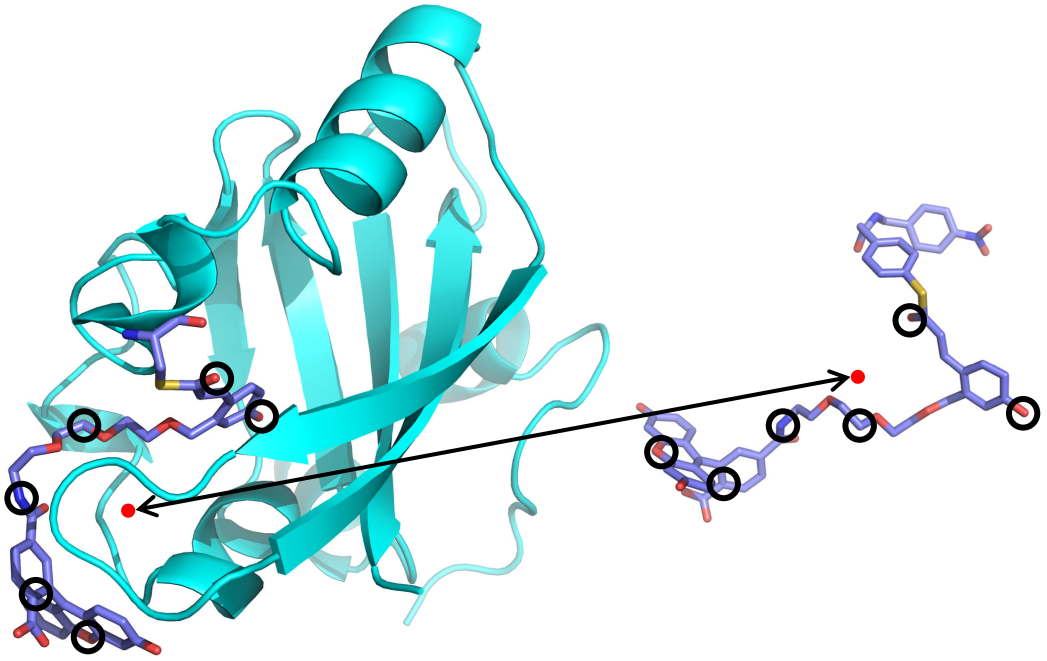

Figure S6. Assessing bound conformations. The figure shows the six heavy atoms used to quantify the degree of binding. Note that in the actual calculation, an ensemble of the 6 final snapshots was used to represent the bound state.

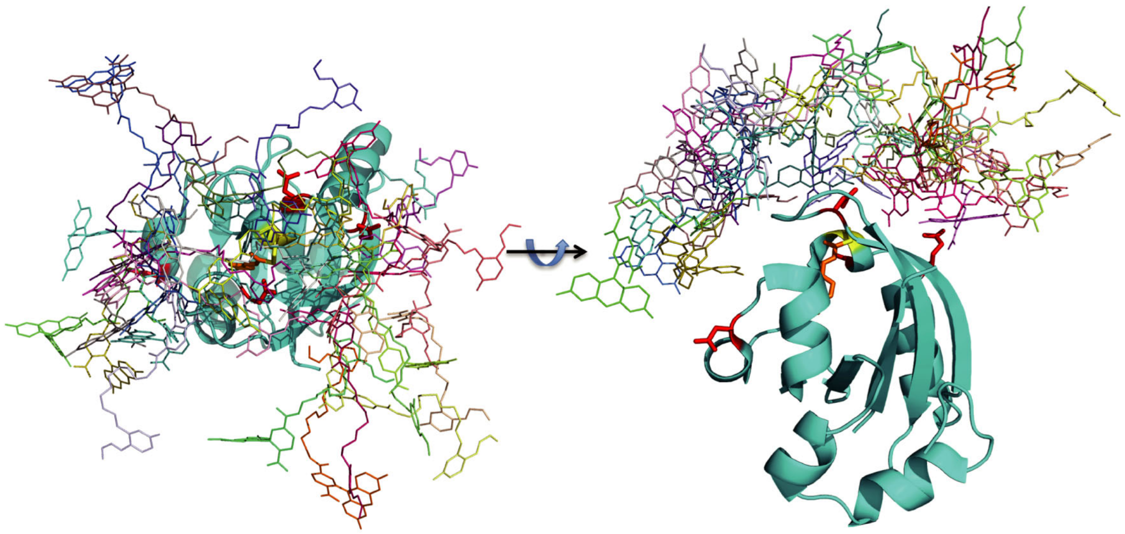

Figure S7. Two views of the initial placement of tFCANB for 30 independent explicit solvent MD simulations.

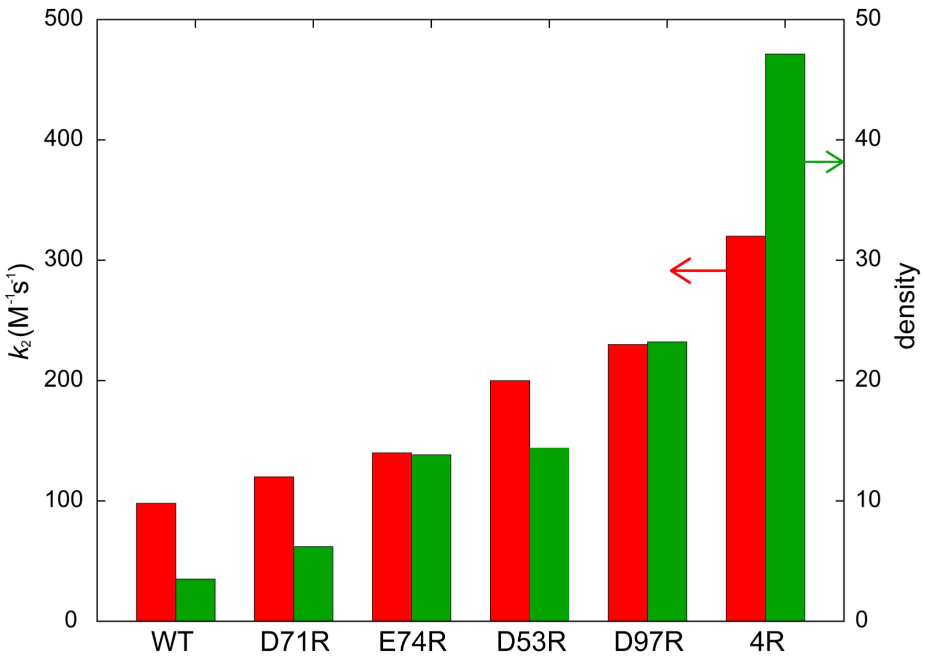

Figure S8. Density of bound states from implicit MD computed from explicit MD snapshots. The density as defined by eqn. 1 using the implicit MD histogram where actual snapshots within the 6A radius have been replaced with explicit MD snapshots of tFCANB. The experimentally observed labeling rates, proportionally scaled, are shown as well.

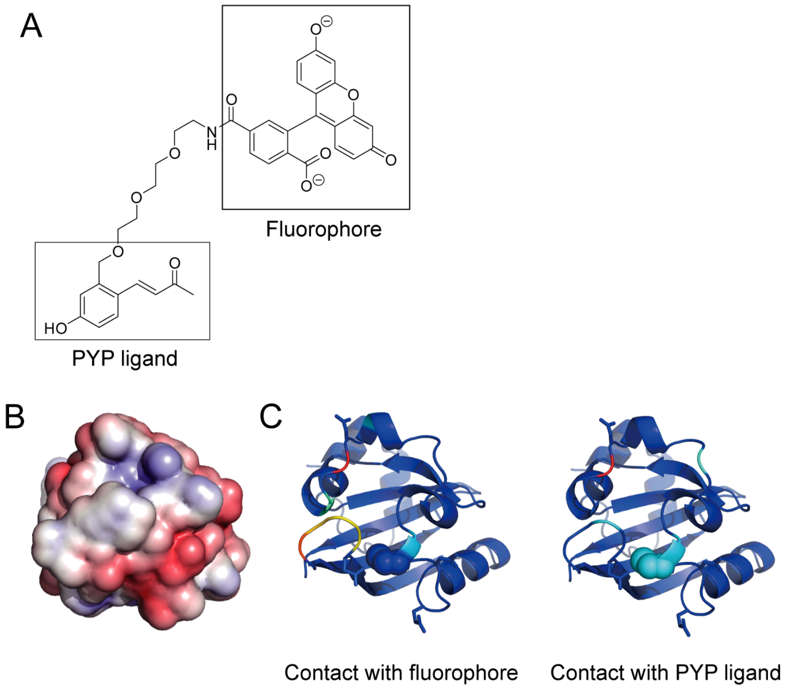

Figure S9. FCANB binding density resulting from the implicit solvent MD simulations.

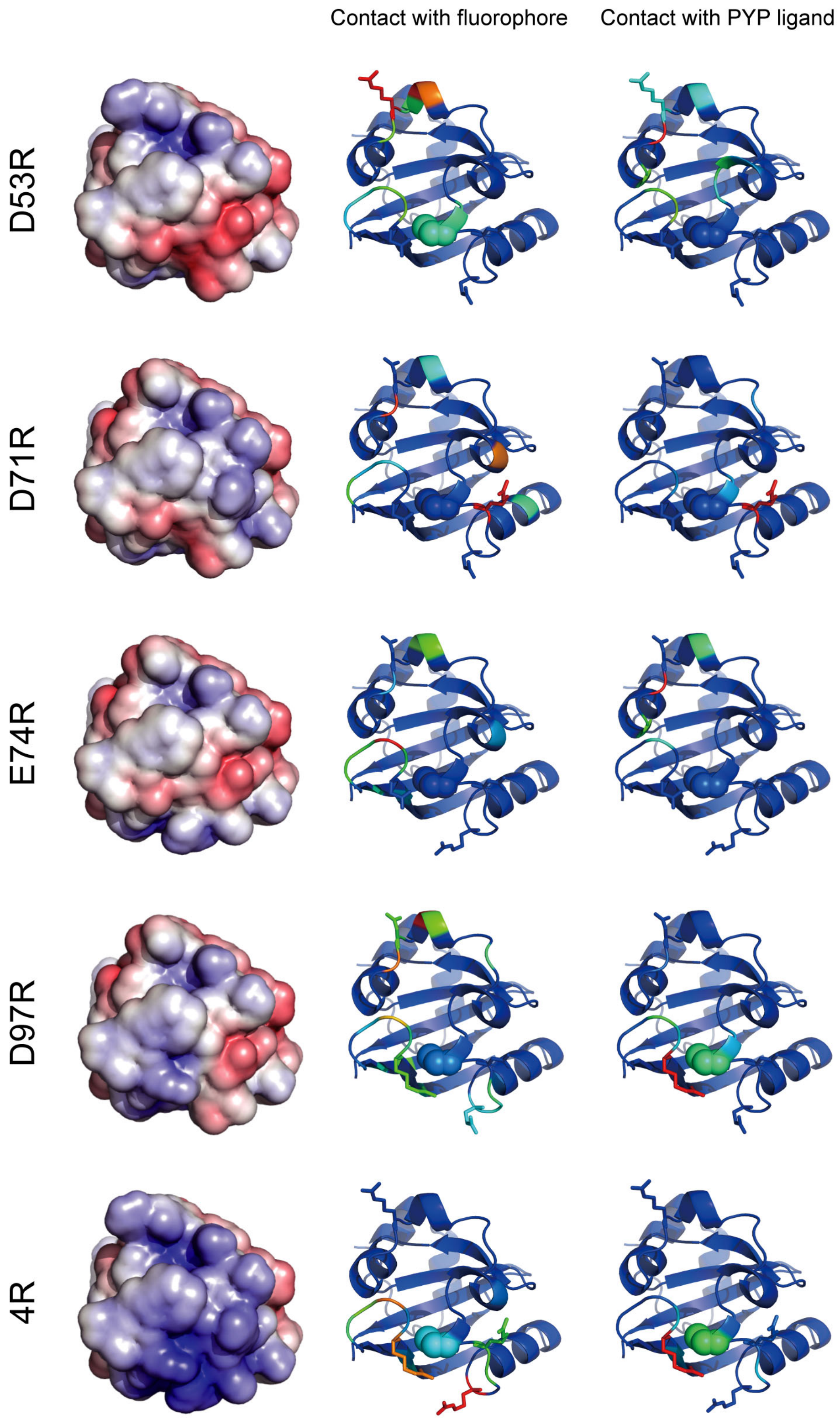

Figure S10. Electrostatic surfaces of the each mutant, along with a heat-map of contacts by the fluorophore and PYP-ligand portions of FCANB.

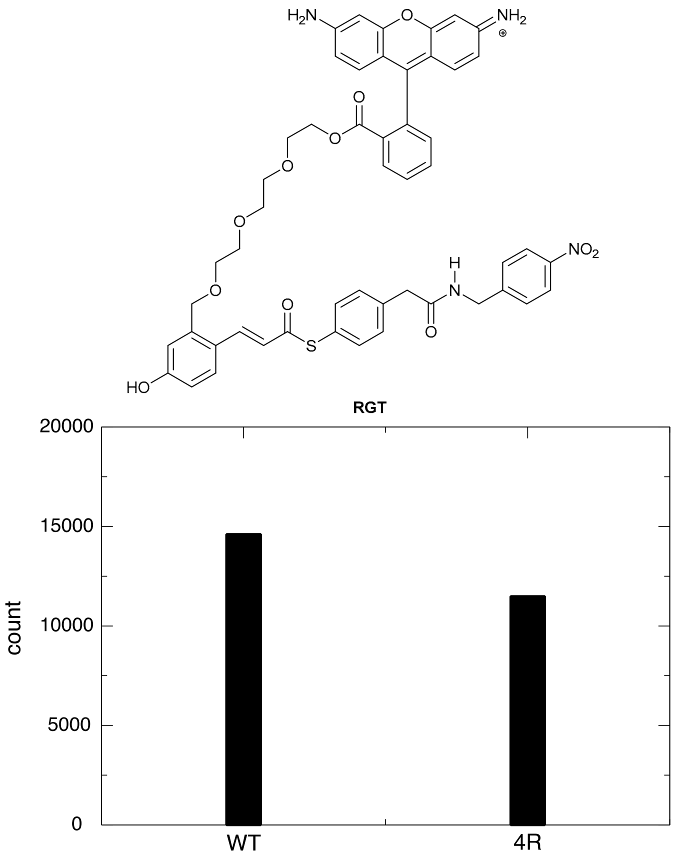

Figure S11. RGT binding density resulting from the implicit solvent MD simulations.

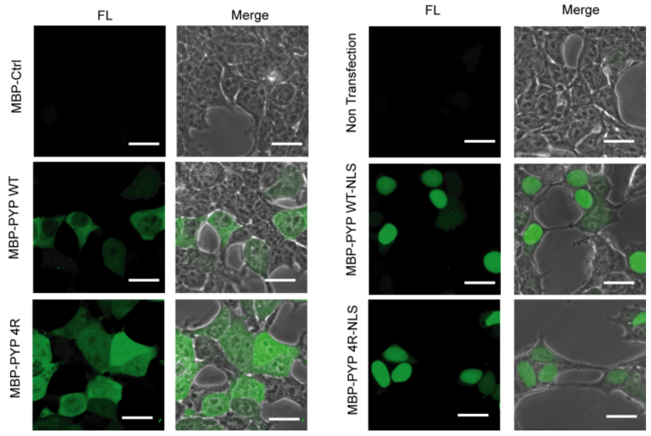

Figure S12. Fluorescent imaging of MBP-PYP (left panels) and MBP-PYP-NLS (right panels) expressing HEK293T cells visualized by AcFCANB. Cells expressing MBP only or Mock (non transfection) were used as controls. AcFCANB was excited at 473 nm and detected at 490-590 nm. Scale bar: 20 μm. Incubation time: 60 min. [AcFCANB]: 500 nM.

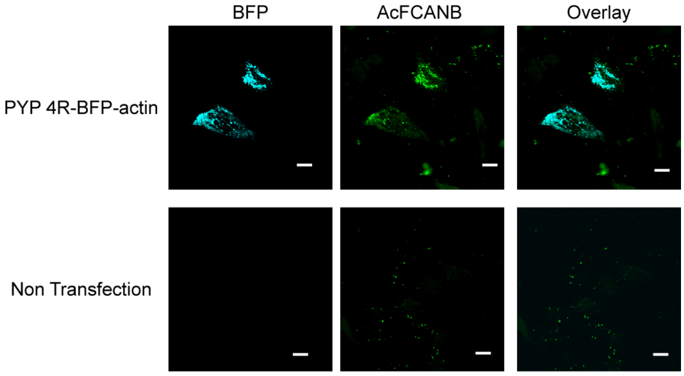

Figure S13. Fluorescent imaging of HeLa cells expressing PYP4R-BFP-actin. Non-transfected cells were used as controls. AcFCANB was excited at 473 nm and detected at 490-590 nm. BFP was excited at 405 nm and detected at 420-520 nm. Scale bar: 20 μm. Incubation time: 30 min. [AcFCANB]: 5 μM.

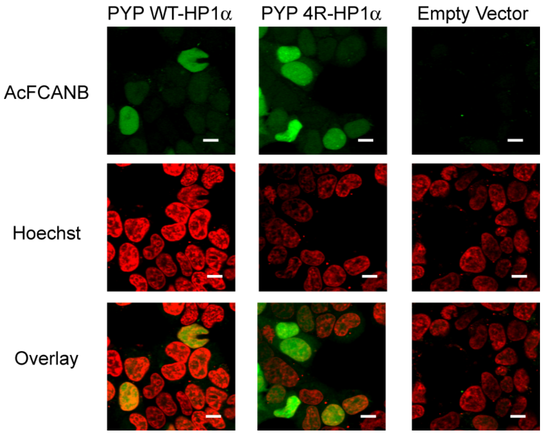

Figure S14. Fluorescent imaging of HEK293T cells expressing PYP-HP1α. Cells expressing Empty Vector (mock) were used as controls. AcFCANB was excited at 473 nm and detected at 490-590 nm. Incubation time: 60 min. [AcFCANB]: 2 μM. Scale bar: 10 μm.

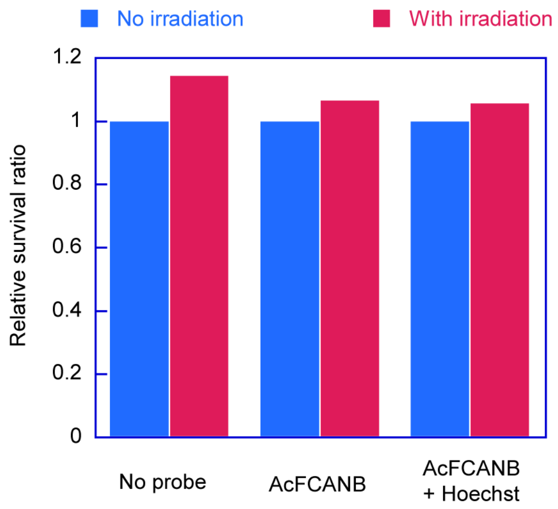

Figure S15. WST assays of HEK 293T cells expressing PYP-HP1α. Cells were irradiated every 15 min over a 150 min-period with the same laser power that was used in the imaging experiments. The cells incubated under a dark condition were used as controls.

Table S1. Calculated pKa values of Cys69-SH.

PYP	pKa
WT	10.11
D53R	10.11
D71R	10.14
E74R	10.11
D97R	9.39
4R	9.41

References

1. Y. Hori, K. Nakaki, M. Sato, S. Mizukami, and K. Kikuchi, *Angew. Chem. Int. Ed.*, 2012, **51**, 5611–5614.

2. Y. Hori, T. Norinobu, M. Sato, K. Arita, M. Shirakawa, and K. Kikuchi, *J. Am. Chem. Soc.*, 2013, **135**, 12360–12365.

3. Y. Hori, H. Ueno, S. Mizukami, and K. Kikuchi, *J. Am. Chem. Soc.*, 2009, **131**, 16610–16611.

4. D. Case, V. Babin, J. Berryman, R. Betz, Q. Cai, D. Cerutti, T. Cheatham, III, T. Darden, R. Duke, H. Gohlke, A. Goetz, S. Gusarov, N. Homeyer, P. Janowski, J. Kaus, I. Kolossváry, A. Kovalenko, T. Lee, S. LeGrand, T. Luchko, R. Luo, B. Madej, K. Merz, F. Paesani, D. Roe, A. Roitberg, C. Sagui, R. Salomon-Ferrer, G. Seabra, C. Simmerling, W. Smith, J. Swails, R. Walker, J. Wang, R. Wolf, X. Wu, and P. Kollman, 2012.

5. B. Hess, C. Kutzner, D. van der Spoel, and E. Lindahl, *J. Chem. Theory Comput.*, 2008, **4**, 435–447.

6. A. da Silva and W. Vranken, *BMC Res. Notes*, 2012, **5**, 367.

7. B. Hess, H. Bekker, H. J. C. Berendsen, and J. G. E. M. Fraaije, *J. Comput. Chem.*, 1997, **18**, 1463–1472.

8. M. Parrinello, *J. Appl. Phys.*, 1981, **52**, 7182–7190.

9. M. H. Olsson, C. R. Sondergaard, M. Rostkowski, and J. H. Jensen, *J. Chem. Theory Comput.*, 2011, **7**, 525–537.
